# Genetic variant in *SLC1A2* is associated with elevated anterior cingulate cortex glutamate and lifetime history of rapid cycling

**DOI:** 10.1038/s41398-019-0483-9

**Published:** 2019-05-23

**Authors:** Marin Veldic, Vincent Millischer, John D. Port, Ada Man-Choi Ho, Yun-Fang Jia, Jennifer R. Geske, Joanna M. Biernacka, Lena Backlund, Susan L. McElroy, David J. Bond, J. Carlos Villaescusa, Michelle Skime, Doo-Sup Choi, Catharina Lavebratt, Martin Schalling, Mark A. Frye

**Affiliations:** 1 Department of Psychiatry and Psychology, Mayo Clinic, Rochester, MN USA; 2 Department of Molecular Medicine and Surgery (MMK), Karolinska Institutet, Stockholm, Sweden; 3 Neurogenetics Unit, Center for Molecular Medicine, Karolinska University Hospital, Stockholm, Sweden; 4 Department of Radiology, Mayo Clinic, Rochester, MN USA; 5 Department of Molecular Pharmacology & Experimental Therapeutics, Mayo Clinic, Rochester, MN USA; 6 Department of Health Sciences Research, Mayo Clinic, Rochester, MN USA; 7 Lindner Center of Hope, University of Cincinnati, Cincinnati, OH USA; 8 Department of Psychiatry, University of Minnesota, Minneapolis, MN USA

**Keywords:** Bipolar disorder, Clinical genetics

## Abstract

Glutamatergic dysregulation is implicated in the neurobiology of mood disorders. This study investigated the relationship between the anterior cingulate cortex (AC) glutamate, as measured by proton magnetic resonance spectroscopy (^1^H-MRS), and single-nucleotide polymorphisms (SNPs) from four genes (*GLUL*, *SLC1A3*, *SLC1A2*, and *SLC1A7*) that regulate the extracellular glutamate in 26 depressed patients with major depressive disorder (MDD; *n* = 15) and bipolar disorder (BD; *n* = 11). Two SNPs (rs3812778 and rs3829280), in perfect linkage disequilibrium, in the 3′ untranslated region of the EAAT2 gene *SLC1A2*, were associated with AC glutamate, with minor allele carriers having significantly higher glutamate levels (*p* < 0.001) in comparison with common allele homozygotes. In silico analysis revealed an association of minor allele carriers of rs3812778/rs382920 with an upregulation of the astrocytic marker *CD44* localized downstream of *SLC1A2* on chromosome 11. Finally, we tested the disease relevance of these SNPs in a large group of depressed patients [MDD (*n* = 458); BD (*n* = 1473)] and found that minor allele carriers had a significantly higher risk for rapid cycling (*p* = 0.006). Further work is encouraged to delineate the functional impact of excitatory amino acid transporter genetic variation on CD44 associated physiology and glutamatergic neurotransmission, specifically glutamate–glutamine cycling, and its contribution to subphenotypes of mood disorders.

## Introduction

There is increasing recognition that glutamatergic dysregulation is implicated in the neurobiology of mood disorders. The evidence base spans animal studies,^1^ postmortem^2^, imaging^3–5^, and pharmacological studies^3–5^, as well as the latest genome-wide association studies in major depressive disorder (MDD)^6^ and bipolar disorder (BD)^7^.

Most of the glutamate functional neuroimaging work in mood disorders has focused on the prefrontal and cingulate cortices, recognizing the anterior cingulate cortex (AC) as a regulator of emotional and cognitive behavior^8^. Magnetic resonance spectroscopy (MRS) is a functional brain imaging method uniquely positioned to investigate glutamatergic biochemical mechanism of action^9,10^. Previous work indicates that glutamate, glutamine, or the composite glutamate/glutamine levels in depression may differ by diagnostic subtype^3,11,12^. While brain regions, magnet strength, glutamate MRS sequence, and post-processing methods differ, available MR spectroscopic evidence to date suggests that glutamate levels are increased in BD and reduced in MDD^11–13^.

**Fig. 1:**
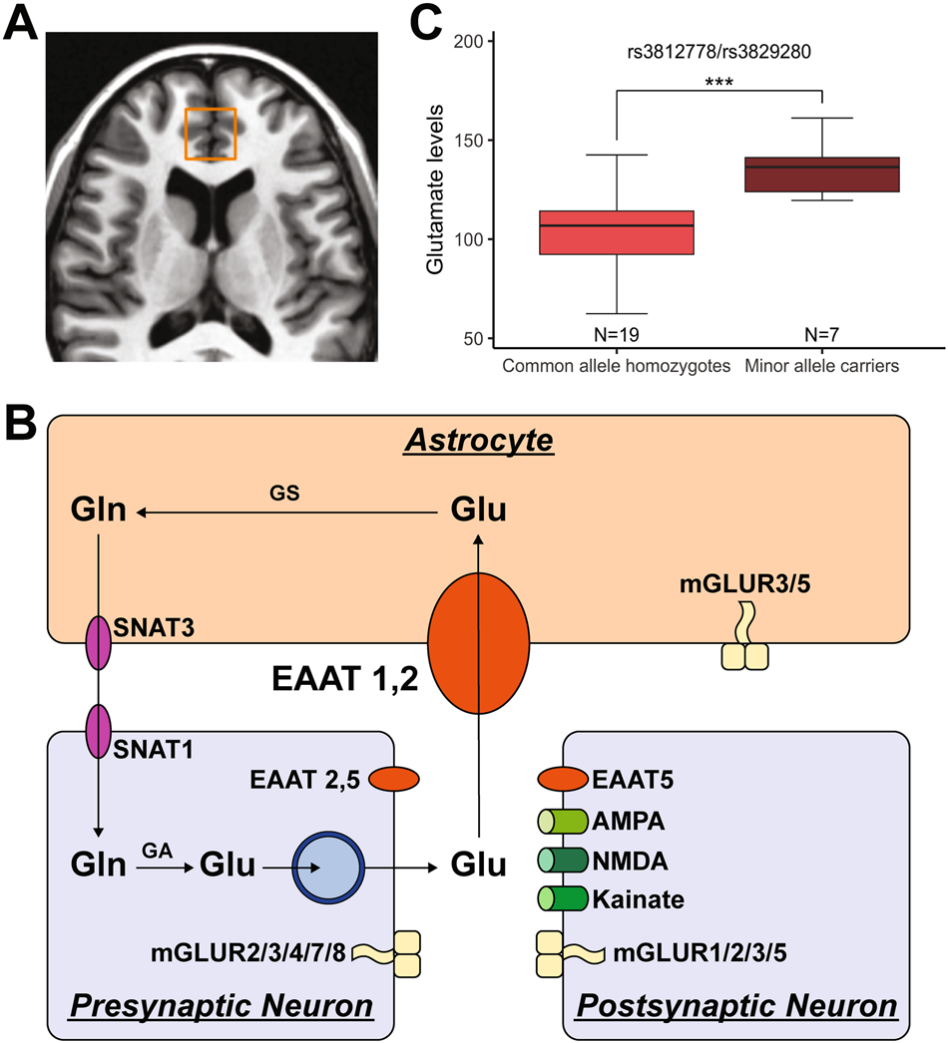
Anterior cingulate cortex glutamate levels in common homozygotes and minor allele carriers for *SLC1A2* single-nucleotide polymorphisms (SNPs) rs3812778/rs3829280. **a** MRI location for the pregenual anterior cingulate cortex ^1^H-MRS voxel acquisition. The reference image of an 8-cm^3^ voxel (2 × 2 × 2 cm) of predominantly (prefrontal) gray matter was centered on the frontal interhemispheric fissure. The posterior margin of the voxel was placed immediately anterior to the genu of the corpus callosum in an area corresponding to the pregenual anterior cingulate cortex (Brodmann area 24a, 24b, and 32). **b** Glutamate–glutamine cycle and glutamate neurotransmission in the anterior cingulate cortex. Glutamate exerts its action on a variety of ionotropic (AMPA, NMDA, Kainate) and metabotropic (mGLUR 1–8) glutamate receptors. Glutamate is transported from the synaptic cleft into astrocytes by excitatory amino acid transporters. In astrocytes, glutamate is converted to glutamine by the astrocyte-specific enzyme glutamine synthetase and shuttled to the presynaptic neuron by sodium-coupled neutral amino acid transporters. In presynaptic neurons, phosphate-activated glutaminase converts glutamine back to glutamate. Glu glutamate, *Gln* glutamine, EAAT excitatory amino acid transporter, SNAT sodium-coupled neutral amino acid transporter, mGLUR metabotropic glutamate receptors, AMPA α-amino-3-hydroxy-5-methyl-4-isoxazolepropionic acid receptor, NMDA N-methyl-D-aspartate receptor, Kainate kainate receptor, GS glutamine synthetase, GA glutaminase, Pre-SN presynaptic neuron Post-SN post-synaptic neuron. **c** Boxplot representations (median, 25th and 75th percentile) of glutamate levels measured by two-dimensional J-resolved averaged PRESS sequence in a combined group of unipolar and bipolar depressed common homozygotes and minor allele carriers for *SLC1A2* SNPs rs3812778 (G/A) and rs3829280 (A/T). ***Homozygotes versus minor allele carriers, *p* = 0.00078 for both SNPs

In this study, in a mixed population of patients with MDD and BD, we evaluated the relationship between ^1^H-MRS glutamate in the AC and single-nucleotide polymorphisms (SNPs) of astrocyte-specific genes, *GLUL*, *SLC1A3*, and *SLC1A2*, encoding for glutamine synthetase (GS), excitatory amino acid transporter (EAAT) 1 and EAAT2, respectively, which are known to regulate synaptic or extracellular glutamate levels in the astrocyte. We also included *SLC1A7*, which encodes for EAAT5, and is co-expressed with *SLC1A2*^14^ (Fig. 1b). Positive hits were followed up in silico. Finally, based on our previous work on rapid cycling (RC)^15,16^, including genetic findings linking RC to glutamate physiology, we investigated the association between positive hits and this phenotype in a large group of unipolar and bipolar depressed patients.

## Materials and methods

### Participants for the MRS study

The MRS study was approved by the Mayo Clinic Institutional Review Board (IRB# 06-006659). Potential subjects were identified and referred to the study by Mayo Clinic psychiatrists and psychologists from inpatient and outpatient services, as well as a general intra campus newsletter. After obtaining written informed consent, 51 individuals, ages 18–65 were diagnosed using the Structured Clinical Interview for DSM-IV (SCID)^17^; this diagnostic interview was administered by trained raters directly supervised by the principal investigator (MAF). The inclusion criteria for this study were a current DSM-IV diagnosis of a major depressive episode associated with MDD, BD I, or BD II, based on SCID, and a negative toxicology screen and pregnancy test. Exclusion criteria included: inability to speak English or provide informed consent, current treatment with an antidepressant, history of active substance abuse within the last 6 months, abnormal thyroid-stimulating hormone, unstable medical illness, Young Mania Rating Scale (YMRS)^18^ > 12 consistent with hypomania, active suicidal ideation with plan, current psychosis, and antipsychotic treatment within 4 weeks.

Depressive and manic symptom severity was assessed with the Hamilton Depression Rating Scale-28 Item Version (HAM-D28)^19,20^, to assess for atypical neurovegetative symptoms, and the YMRS respectively. All ratings were conducted by the principal investigator (MAF) or inter-rater-reliable assistants.

### Participants for the genotyping cohort

The cohort consisted of patients with BD and MDD. The BD cohort consisted of patients from the Mayo Clinic Individualized Medicine Biobank for Bipolar Disorder (IRB# 08-008794)^21^ and patients recruited from the Unit of Affective Disorders, Psychiatry Southwest, Karolinska University Hospital, Huddinge, Stockholm, Sweden^16^. The assessment was based on interviews, medical records, and questionnaires and performed by specialized psychiatrists or by trained psychiatric nurses. Patients with MDD were selected from the PART study^22^, a longitudinal population-based study in Stockholm County, Sweden, utilizing the Major Depression Inventory (MDI)^23^.

Rapid cycling has been identified by the biobank as a clinical phenotype to further investigate the underlying genetics and neurobiology^15^. Lifetime history of rapid cycling (RC) was defined as a self-reported history of having four or more distinct bipolar mood episodes in a 12-month period, with each episode separated by a return to baseline mood state for at least 2 months, or a switch to the opposite mood pole. Manic and hypomanic episodes were counted as being on the same mood pole.

Further description of cohort including clinical variables quantified can be found in the Supplementary Information.

### MR imaging (MRI) and 1H-MRS acquisition

Imaging and acquisition was completed with a GE 3T Discovery 750 MRI scanner with 22.1 software and an 8-channel head coil by a neuroradiologist blinded to the group allocation throughout the entire study who did not participate in assessing the outcome. The axial plane was landmarked in all subjects at the center of the forehead, 1 cm above the eyebrows to standardize head position from scan to scan. A neuroradiologist reviewed baseline and posttreatment structural MRI data for potential exclusionary head and brain pathology.

A FAST 3D SPGR sequence was used to acquire volumetric data for cerebrospinal fluid (CSF) correction (axial acquisition; repetition time [TR] = 12.6 ms, echo time [TE] = 5.6 ms, flip angle = 15°, voxel dimensions = 0.49 × 0.49 × 1.5 mm). Voxel positioning for the midline anterior cingulate cortex (MACC) and for the left dorsolateral prefrontal cortex (LDLPFC) voxels followed a systematic approach during all scans (Fig. 1a; Supplementary Information).

Based on the prior literature^24,25^, we chose two different 1H-MRS sequences for our glutamate and glutamine measurements, each with its own strengths. A TE-optimized PRESS sequence was used to measure both glutamate and glutamine (PROBE-P PRESS; TE = 80 ms, TR = 2000 ms, no. of excitations = 8, no. of acquisitions = 128)^25^. A two-dimensional J-resolved averaged PRESS sequence was used with the goal of collecting an optimized measure of glutamate (2DJ PRESS; TE = 35–195 ms in 16 steps, TR = 2000 ms, excitations = 8)^26,27^.

### Reconstruction and quantification of spectra

Spectroscopic imaging data were transferred to a Sun workstation running SAGE-IDL (GE Medical Systems). The data integrity was verified visually; scans with artifact were excluded from the study. A quantitative analysis of brain metabolites was performed using the LC Model software. Basis sets for both the 3T-PRESS and 3T-2DJ were provided by the vendor. The lower bound of measurement error for glutamate quantification was a Cramer–Rao lower bound of 20 or less. For glutamine quantification, the lower bound measurement error was relaxed to 30 or less to optimize both limited data and goodness of fit^28,29^.

The SPGR anatomical data were segmented into gray matter, white matter, and CSF using a technique modified from a previous study^30^ revised to use the FSL package from FMRIB Oxford^31^. Briefly, SPGR data were converted into NIFTI format using mri_convert. The T1 volume was skull-stripped using BET, then segmented into gray matter, white matter, and CSF using FAST with default parameters. The segmented data were then overlaid with the voxel location using in-house software, and the number of pixels of each tissue type within the voxel was counted. These counts were then normalized to the total number of pixels within the voxel to arrive at the fraction of each tissue within the 1H-MRS voxel. The tissue volume-corrected metabolite concentrations, [M]TVC, were then calculated by taking the measured metabolite concentration, [M]M, and applying a correction factor as follows: [M]TVC = [M]M x (1/[1−FCSF]) where FCSF is fraction of CSF. This generated “absolute” (vs relative to creatine) metabolite concentrations in “institutional units” specific to our scanner and technique. These CSF-corrected metabolite concentrations were used for all statistical analyses.

AC and DLPFC MRS data acquisition of both TE80 and 2DJ Press, spectra reconstruction and quantification were successfully completed in 39 individuals (BD: *N* = 18, UD: *N* = 21); remaining subjects were either screening failures or MRS was of a poor quality (i.e., inadequate Cramer–Rao bound, head movements during data acquisition).

### Genetic analysis of the MRS cohort

Of the 39 MRS-examined individuals, 26 subjects consented to a blood draw for genetic analysis. Prior to study initiation, we designated 16 SNPs located in essential regulatory elements and coding sequences of *GLUL* (2 SNPs), *SLC1A3* (1 SNP), *SLC1A2* (12 SNPs), and *SLC1A7* (1 SNP). We amplified genomic DNA regions containing targeted SNPs and sequenced amplicons using an ABI 3730xl automated sequencer (Applied Biosystems, Foster City, CA, USA). Sequence variants were then analyzed by Mutation Surveyor version 2.2 (Softgenetics, PA). One *SLC1A2* SNP, rs12360706, was excluded from the analysis due to poor sequencing quality **(**Supplementary Table 1**)**. Three groups in perfect LD (R^2^ = 1) could be determined: (1) rs1043101, rs10768121, rs11033046, rs12361171, and rs3088168; (2) rs3812778 and rs3829280; (3) rs10742338 and rs2229894, leaving nine independent (R^2^ < 0.6) loci.

### In silico analyses

LDlink (https://analysistools.nci.nih.gov/LDlink/) was used to perform proxy search for SNPs in LD with rs3812778, using populations of European descent. Expression quantitative trait loci (eQTL) were identified in the DLPFC using the gene expression database BrainCloud (http://braincloud.jhmi.edu/)^32^, based on RNA sequencing and genotype data of 412 subjects. The modeling tested for additive genetic effects on expression, adjusted for sex, ancestry, and expression heterogeneity. A SNP-feature pair was considered significant with a false discovery rate less than 1%. Raw data for the significant pair were obtained from the website. Furthermore, data were obtained for *CD44* from the UK Brain Expression Consortium (UKBEC) (http://www.braineac.org/), which includes microarray data and genetic markers from different brain regions from 134 subjects. Genomic annotations were used from UCSC genome for histone modifications and DNAseI-sensitive regions^33^. The development transcriptome dataset summarized to genes from the BrainSpan project (http://www.brainspan.org/)^34^ was used to assess correlations between expression of *CD44* and several genes of interest. This data set contains RNA-sequencing data from up to sixteen brain regions from 42 donors across the full course of human brain development. SNPs were functionally annotated using the genome-wide annotation of variants (GWAVA) tool, which supports prioritization of noncoding variants by integrating various genomic and epigenomic annotations (https://www.sanger.ac.uk/science/tools/gwava)^35^.

### Genetic analyses of the genotyping cohort

DNA samples from peripheral blood collected in Sweden and at the Mayo Clinic were genotyped for the SNPs rs3812778 and rs3829280 in *SLC1A2* using TaqMan SNP genotyping assays on QuantStudio 7 Flex instrument (Applied Biosystems, Foster City, CA, USA). The genotyping was performed by an investigator blinded to the disease status of the patients. The genotyping efficiency was 98%.

### Statistical analysis

Normality was assessed with quantile–quantile plots, homogeneity of variance was tested using the Levene’s test.

Demographic and clinical measures are presented using descriptive statistics. Comparisons between MDD and BD groups were made using *t* tests for continuous measures and a chi-square test for sex.

Linear regression models were used to test the additive effect of the minor allele (coded as 0, 1, 2) on midline AC and LDLPFC glutamate concentration for each SNP, followed by a two-sided *t* test in a dominant model when the number of minor allele homozygotes was low (i.e., grouping A/G and A/A for rs3812778, and A/T and T/T for rs3829280). A Bonferroni correction was applied for 36 (nine loci, two regions, two methods) independent tests (*p*_cor_). Two-sided *t* tests were used to test for differences in glutamate levels between BD and MDD.

The association between *CD44* expression and the genetic data was tested by two-sided *t* test using a dominant model. In the UKBEC data set, q-values were used to estimate false discovery rates (FDR). Correlations between the logarithm of *CD44* expression and the logarithm of the expression of the genes of the glutamate–glutamine cycle were assessed using Spearman correlation coefficient.

Differences in genotype between diagnoses, as well as between RC BD and non-rapid cycling (NRC) were tested using chi-square, as well as logistic regression to correct for sex and age. A Bonferroni correction for two independent tests was applied (*p*_cor_).

Statistical analyses were conducted using SAS (version 9.4; Cary, NC) and R programming language.

## Results

### rs3812778/rs3829280 are associated with AC glutamate levels

As presented in Table 1, there was no statistically significant difference for age (*p* = 0.075), sex (*p* = 1.0), or mood symptom severity, as measured by HAM-D28 (*p* = 0.073) between mood disorder subtypes.

**Table 1:** Subject demographics

	MR spectroscopy—genotyping		Genotyping		
	BD	MDD	Rapid cycling BD	Non-rapid cycling BD	MDD
Number of participants^1^	11	15	638	835	458
Age (mean ± SD)	33 (11.3)	35.2 (13.3)	41.2 (14.2)	47.2 (15.4)	51.8 (11.9)
Sex (female/male)	8/3	11/4	403/235	463/372	330/128
HAM-D (mean, SD)	36.3 (9.1)	29.9 (7.7)	NA	NA	NA

*BD* bipolar depression, *MDD* major depressive disorder, *NA* not applicable

^1^All participants were of Swedish or Caucasian American origin

The minor alleles of the two SNPs rs3812778 and rs3829280 (in perfect linkage disequilibrium (LD, *r*^2^ = 1) in the 3′ UTR region of *SLC1A2* gene) were associated with elevated 2D JPRESS mean AC glutamate levels (common allele homozygotes: 105 ± 21 units, minor allele carriers 135 ± 15 units; *p* *=* 0.00078, *p*_cor_ = 0.028) (Fig. 1c). No association between glutamate levels and diagnosis (*p* *=* 0.68), or depression symptom severity (*p* = 0.75) was found. There was no association between glutamate levels and any other SNP. There was also no association between any SNPs, including rs3812778/rs3829280, when combined glutamate/glutamine levels were analyzed using the TE80 method. No association was found in the LDLPFC (Supplementary Table 1).

### rs3812778/rs3829280 are associated with CD44 levels

**Fig. 2:**
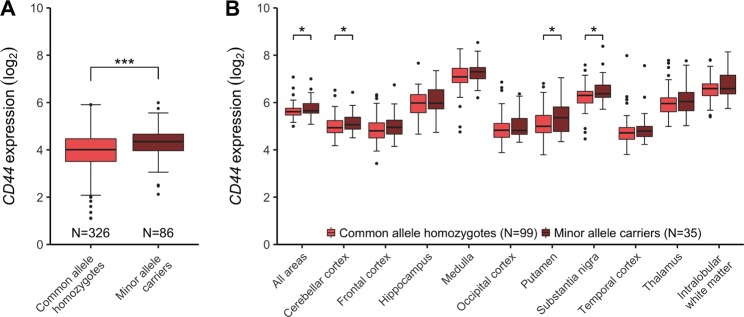
Expression of *CD44* in different brain regions. Boxplot representations (median, 25th and 75th percentile) of *CD44* expression stratified by rs3812778/rs3829280 **a** *CD44* (chr11:35240935-35243200(*)) in the dorsolateral prefrontal cortex as measured by RNA sequencing (data from BrainCloud), (**b**) *CD44* (Affimetrix transcript t3326635) in ten different brain regions, measured by microarray (data from the UK Brain Expression Consortium). ***p = 0.00010, *p < 0.05

In silico analyses, using the BrainCloud eQTL-browser, we found an association between the minor allele of rs3829280 and higher levels of the *SLC1A2* neighboring gene *CD44* mRNA (chr11:35240935-35243200(*)) (Fig. 2a, *p* *=* 0.00010). These findings were strengthened with data from the UK Brain Expression Consortium, where significant associations were identified between the minor allele of rs3812778/rs3829280 and higher levels of the full-length transcript of *CD44* in the cerebellar cortex, putamen, and substantia nigra, as well as in the average of all measured brain regions (Fig. 2b). Significant associations were also found for other *CD44* transcripts (Supplementary Table 2). When searching for potential surrounding functional SNPs, we found six SNPs in perfect LD (R^2^ = 1) with rs3812778/rs3829280: rs10836358, rs67384276, rs56193087, rs1570216, rs4508184, rs12360706. Analysis with GWAVA, a tool for functional annotation of noncoding sequence, revealed high values across all prediction scores (> 0.6) for rs1570216, indicating high probability for functionality for this SNP lying in the 3′-UTR of *SLC1A2* in a genomic area sensitive for DNaseI also rich in H3K27 acetylation and H3K4 monomethylation (Supplementary Table 3).

### CD44 strongly correlates with astrocytic markers

**Fig. 3:**
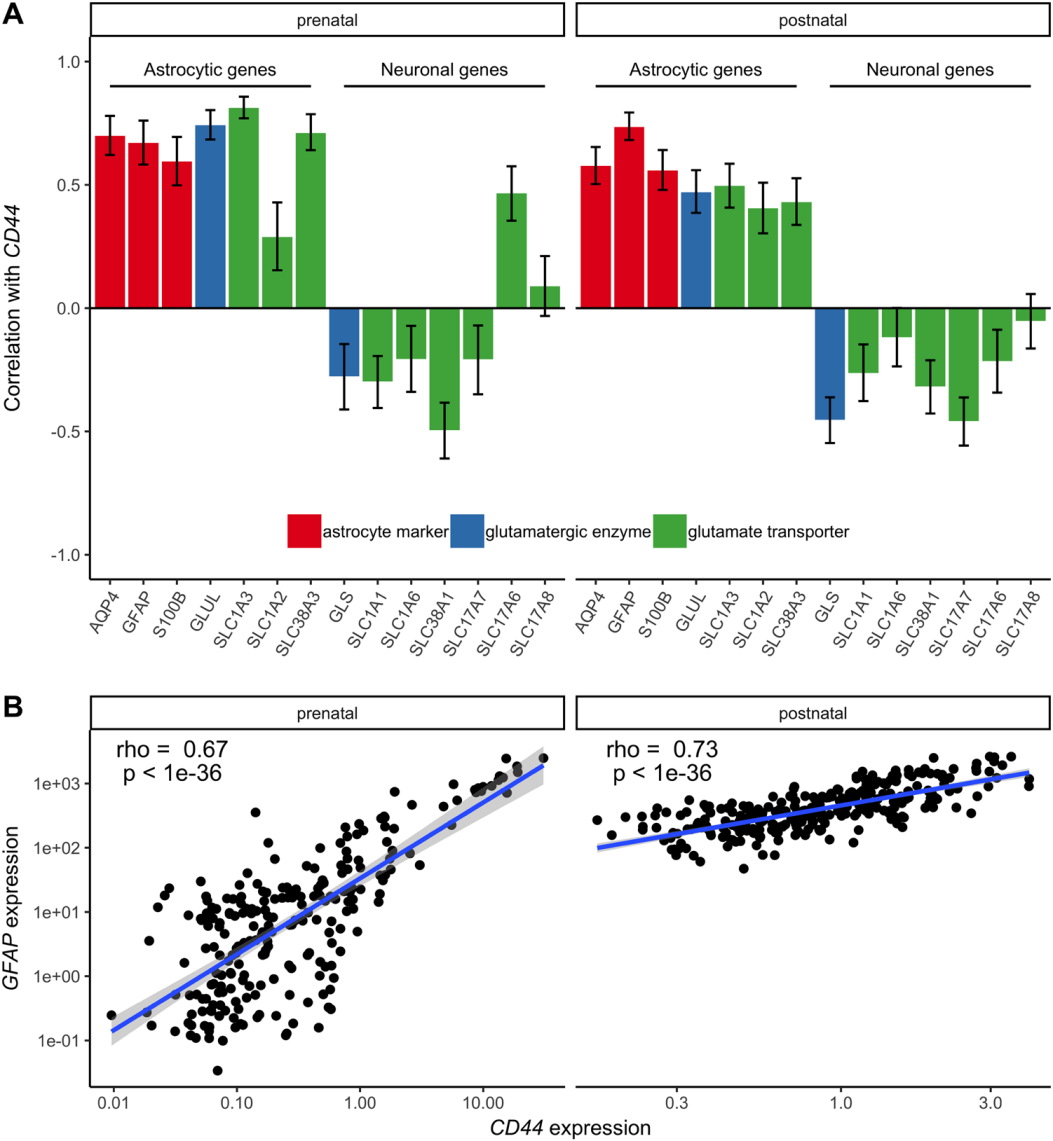
Correlations between *CD44* expression and genes of the glutamate/glutamine cycle across several brain regions pre- and postnatally. **a** Correlations between *CD44* expression (log) and the genes of the glutamate/glutamine cycle (log) across several brain regions in prenatal brains (*N*_donors_ = 20, *N*_datapoints_ = 237) and postnatal brains (*N*_donors_ = 22, *N*_datapoints_ = 287), reported as Spearman’s correlation coefficients (error bars 95% CI). **b** Spearman correlation between the expression of *CD44* and the astrocytic marker *GFAP* in pre- and postnatal brains

Given the important role CD44 plays in brain development^36^, the correlation between *CD44* expression and the expression of genes of the glutamate–glutamine cycle were assessed across several brain regions pre- and postnatally using data from the BrainSpan project^34^ (Fig. 3a). Strong positive correlations between *CD44* and genes typically expressed in astrocytes (*GLUL, SLC1A3, SLC1A2*, and SLC38A3) were seen both pre- and postnatally, while no or negative correlations were observed for genes typically expressed in neurons (*GLS, SLC1A1, SLC1A6, SLC38A1, SLC17A7, SLC17A6, SLC17A8*, and *SLC1A7*). Furthermore, *CD44* also very strongly correlated with typical astrocytic markers like *AQP4, S100b*, and *GFAP* (Fig. 3b).

### rs3812778/rs3829280 and rapid cycling (RC) prevalence

We then performed an exploratory investigation to check for disease relevance. Similar to the genetic spectroscopic study, the follow-up cohort was composed of both MDD and BD individuals of Swedish and Caucasian American origin. Demographic characteristics can be found in Table 1. rs3812778/rs3829280 were in Hardy–Weinberg equilibrium, and the minor allele frequencies (MAF) of both SNPs in the whole cohort were 13%, corresponding to those in European populations^37^.

There was no significant difference in the percentage of minor allele carriers of rs3812778/rs3829280 in BD vs MDD participants (odds ratio (OR): 1.05 [95% confidence interval (CI): 0.84–1.32], *p* = 0.647). While the percentage of minor allele carriers was comparable between MDD (21.9% [95% CI: 19.1–24.9]) and non-RC (NRC) BD (21.8% [95% CI: 17.9–25.8]), RC BD participants had a significantly higher percentage of minor allele carriers in comparison with the MDD+NRC BD group (26.9% [95% CI: 23.5–30.5]) (age- and sex-adjusted OR: 1.38 [95% CI: 1.09–1.73], *p* = 0.006, *p*_cor_ = 0.012; unadjusted OR: 1.32 [95% CI: 1.05–1.64], *p* = 0.015, *p*_cor_ = 0.03).

**Fig. 4:**
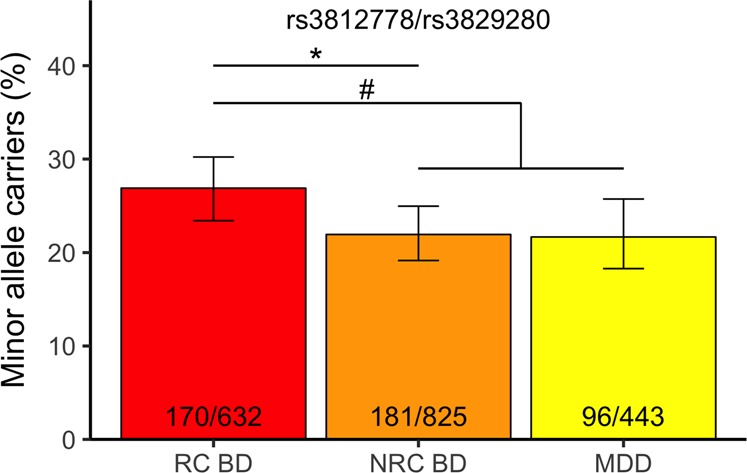
Percentage of rs3812778 and rs3829280 minor allele carriers in patients with rapid cycling BD, non-rapid cycling BD and major depressive disorder. Bar graph representation of the percentage (error bars: 95% CI) of minor allele carriers (rs3812778: A/G and G/G; rs3829289: A/T and T/T) in the different diagnostic groups. *Patients with rapid cycling BD versus patients with non-rapid cycling BD, chi-square test, *p* = 0.028; ^#^patients with rapid-cycling BD versus combined patients with non-rapid cycling BD and MDD, chi-square test, *p* = 0.015. RC BD rapid-cycling BD, NRC BD non-rapid cycling BD, MDD major depressive disorder

Focusing only on patients with BD, a similar effect could be observed between RC BD and NRC BD (age- and sex-adjusted OR: 1.37 [95% CI: 1.07–1.76], *p* = 0.012, *p*_cor_ = 0.024; unadjusted OR: 1.31 [95% CI: 1.03–1.67], *p* = 0.028, *p*_cor_ = 0.056; Fig. 4). The lifetime history of rapid cycling was higher (58%) in the American sites (tertiary referral clinic) than at the Swedish site (a primary referral site, 28%). The model was therefore corrected for site, without significantly affecting the outcome (age, sex, and site-adjusted OR: 1.40 [95% CI: 1.08–1.82], *p* = 0.011, *p*_cor_ = 0.22).

## Discussion

Here, we report a significant association between the minor alleles of rs3812778/rs3829280, two SNPs in perfect linkage disequilibrium in the 3′ UTR of the EAAT2 gene *SLC1A2*, and 2DJ glutamate levels in the AC. After being released from presynaptic nerve terminals, the extracellular glutamate is cleared by a family of excitatory amino acid transporters (EAAT1-5)^38^ (Fig. 1b). Astrocytes play a major role in glutamate homeostasis in the neocortex, with EAAT2, the most abundant glutamate transporter in the forebrain, responsible for up to 95% of glutamate clearance in the mammalian brain, mainly being expressed on astrocytic plasma membranes.^39,40^

Glutamate levels in the AC have been associated with mood disorders. There has been previous speculation that glutamate levels may distinguish BD (i.e., increased glutamate) from MDD (i.e., decreased glutamate)^12,13,41^. However, glutamate levels in the brain are also regulated by genetic variations in molecules, such as EAAT and enzymes responsible for glutamate–glutamine conversion and glutamine–glutamate conversion which have not been studied comparatively in different types of mood disorders. For instance, Ongur et al.^42^ showed that a specific haplotype of four SNPs within *GLS1*, the gene encoding for the enzyme glutaminase generating glutamate from glutamine, was significantly associated with glutamine/glutamate in the parietooccipital cortex and rs956572 in a mixed group of healthy controls and patients with bipolar disorder and schizophrenia^42^. A second example is the work that identified a SNP in B-cell lymphoma 2 (Bcl-2) shown to be associated with increased anterior cingulate cortical glutamate solely in euthymic bipolar I disorder^43^.

In silico analysis showed that the minor allele of rs3812778/rs3829280 was associated with increased levels of *CD44* mRNA. *CD44* is situated downstream of *SLC1A2* on chromosome 11 (Supplementary Fig. 1a) and codes for a transmembrane glycoprotein acting as a receptor for hyaluronan, a key component of the extracellular matrix in the brain. It is implicated in cell-matrix binding, signaling, and cell migration^44^, as well as in the activation and the resolution of inflammatory processes^45^ and plays important roles in physiology (e.g., organogenesis) and pathology (e.g., cancer and metastasis)^44^. In the CNS, *CD44* is mainly expressed on glial cells, in particular astrocytes, but expression has also been shown on neurons^36^, in neural stem cells, astrocyte, and oligodendrocyte precursor cells at early postnatal stages^46^. *CD44* has been implicated in many physiological CNS functions, such as neural development, axon guidance, and astrocyte differentiation^36^. In humans, CD44 has been suggested as a candidate gene associated with BD using convergent functional genomics^47,48^. Furthermore, *CD44* has been identified in a brain GWAS study as a possible risk gene for suicidal behavior^49^ and the CD44 ligand hyaluronic acid was reported to be elevated in the CSF of suicide attempters, correlating with blood-brain barrier permeability, a hallmark of neuroinflammation^50^. Higher levels of *CD44* were also reported in the white matter of patients with multiple sclerosis^51^ and astrocytes of patients with Alzheimer’s disease^52^. *CD44* has also been associated with disorders of the CNS in animal models: while *CD44* deficiency is protective against cerebral ischemia injury in mice^53^, *CD44* levels have also been shown to be changed by omega-3 fatty acid treatment in female, but not male mice a stress-reactive knockout animal model of bipolar disorder and co-morbid alcoholism^54^. Finally, an involvement of *CD44* in synaptic transmission has been suggested with Matzke et al. showing that *CD44*-deficient mice had markedly reduced glutamatergic synaptic excitation^55^. Taken together, this evidence points toward a central role of *CD44* in CNS functions, and it can therefore be hypothesized that a disturbance in *CD44* signaling can lead to a change in glutamate turnover.

The strong positive correlations between *CD44* and astrocytic markers, as well as with the genes of the glutamine–glutamate cycle expressed in astrocytes could be indicative of an effect of rs3812778/rs3829280 on astrocyte numbers. The changes in glutamate metabolism could therefore also be explained by changed levels of astrocytic glutamate transporters, including *SLC1A2*. Therefore, we cannot exclude that rs3812778/rs3829280 also affect the expression of *SLC1A2*, but that this effect cannot be detected in brain homogenates. Indeed, *SLC1A2* is highly regulated with various transcription factor-binding sites, as well as regulatory elements in the UTRs^1,56^. EAAT2 has been implicated in the pathophysiology of several disorders of the CNS, including Parkinson’s disease, epilepsy, amyotrophic lateral sclerosis, Alzheimer’s disease, addiction, schizophrenia, as well as MDD and BD.^1^ On the molecular level, there is strong evidence of downregulation of EAAT2 in diverse brain regions in MDD^2,57^. Early stress impact on the gray matter has been shown to be influenced by a functional polymorphism in EAAT2 in BD in the hippocampus, a brain region with greater atrophy in BD versus MDD^58^; this gene-by-environment interaction in the hippocampus has not been described in MDD^59^. Also, epigenetically mediated effects of early-life stress and addiction on EAAT2 expression regulation may play an important role in determining glutamate clearance rates and subsequent in vivo glutamate measurements^60^. Finally, EAAT2 also affects synaptic transmission, as blocking it with dihydrokainate, a specific inhibitor for EAAT2, leads to extended N-methyl-D-aspartate (NMDA)-receptor-mediated excitatory post-synaptic currents^61^.

The regulatory potential of rs3812778/rs3829280 is supported by the fact that these SNPs are in perfect LD with rs1570216, also situated in the 3′ UTR of *SLC1A2*, in a region sensitive for DNaseI, with high H3K27 acetylation and H3K4 monomethylation, pointing toward an active regulatory area. The results from GWAVA, an annotation tool for noncoding variants that integrates various genomic and epigenomic variables, also point toward the functionality of rs1570216^35^.

Our group has previously shown associations between RC and rs2230912, a genetic variation in *P2RX7*^16^, encoding for P2X purinoreceptor 7, a ligand-gated non-selective cation channel, which has also been implicated in modulating glutamatergic signaling^62^. Reporting a novel association between the minor alleles of rs3812778/rs3829280 and an increased risk for RC, we decided to test whether we could find an interaction of the two genetic variants in our cohort and included rs2230912 in our model. However, we did not see an interactive effect between both SNPs, and the effect of rs3812778/rs3829280 on RC was not changed by this additional variable (Supplementary Table 4).

Summarizing our findings, we hypothesize that the minor alleles of rs3812778/rs3829280 are associated with an upregulation of CD44, possibly indicative of an increase in astrocyte numbers in the brain which in combination with excitatory amino acid transporter modulation, is associated with an increased glutamate recycling resulting in dysregulated glutamatergic neurotransmission, associated with an increased risk of RC (Supplementary Fig. 1b). This hypothesis is supported by findings from Michael et al. who has shown that elevated glutamate/glutamine in the DLPFC of BD II patients is associated with RC^63^. To what extent anti-glutamatergic mood-stabilizing anticonvulsants such as lamotrigine, which has an evidence base in treating rapid cycling bipolar II disorder, could impact this interaction remains to be investigated^64^. One can therefore question whether the currently reported differences seen in functional imaging between BD and MDD, are being driven by currently established diagnostic criteria (i.e., presence of absence of a history of hypo/mania) or rather by clinical subphenotypes like the presence of RC^63^, psychosis^65^, or melancholic vs non-melancholic depression subtypes^3^.

### Limitations

An important limitation of our study is the small sample size of the MRS study, and replication in a larger sample is warranted. Furthermore, we only analyzed a small number of genes. Examining additional genes known to be implicated in depression and involved in either the glutamate/glutamine cycle (e.g., GLS1), regulation of neuronal plasticity and cellular resilience (e.g., BCL2), or purinergic signaling (e.g., P2RX7) may provide a better understanding of the underlying neurobiology of these glutamate-level alterations^66–68^. In addition, no experimental evidence proves that the glutamine/glutamate ratio directly reflects synaptic neurotransmission of glutamate. However, J-resolved MRS sequence is optimized for glutamate detection. This sequence attempts to address some major challenges, including resolving glutamine and glutamate signal from underlying macromolecule resonances as well as those from glutamate-conjugate compounds, such as glutathione. Moreover, studying the glutamine/glutamate ratio has been fruitful, and several lines of evidence reviewed above indicate that changes in glutamine/glutamate correlate with and thus are a measure of changes in glutamatergic activity^42^. Another limitation is heterogeneity of diagnostic assessment between BD and MDD. BD assessment was based on interviews, medical records review, and questionnaires in a clinical sample while MDD cases in the genotyping cohort were selected in a random population cohort and defined by MDI, However, validation studies for the use of MDI in making DSM-IV-based diagnosis of depression have been performed in population-based settings^23^, clinical settings^69^, and outpatient settings^70^. In addition, a population-based sample may reduce the effect of confounders, such as propensity toward help seeking. Also, given that the nature of our cohorts, the sample size of our genotype cohorts was fixed. However, power calculations showed that, given our sample size and the allelic frequencies, we would be able to detect, with a power of 80%, an effect size corresponding to an OR of 1.36 for RC versus NRC and 1.4 for the comparison of MDD versus BD. Finally, inter-rater reliability assessment was not conducted between Swedish and American sites.

## Conclusion

This study is the first to associate spectroscopic findings with gene variants in molecules central to glutamate processing in mood disorders. Future studies combining neuroimaging, genotyping, epigenetic, and possibly other quantifiable diagnostic measurements, with deep clinical phenotyping may provide enough elements to construct nosological categories, and invest in developing biological psychiatric phenotypes that can contribute to diagnostic classification and treatment intervention^3,11,71^.

## Supplementary information

Supplementary Material
