# Supplementary material for: Genetic variant in SLC1A2 is associated with elevated anterior cingulate cortex glutamate and lifetime history of rapid cycling
Source: Transl Psychiatry. 2019 May 23;9:149. doi: 10.1038/s41398-019-0483-9 (PMC6533282; doi:10.1038/s41398-019-0483-9)
Supplement: Supplementary file 1 — Supplementary Material [file 41398_2019_483_MOESM1_ESM.pdf]

Supplementary Data for

Genetic Variant in *SLC1A2* is Associated with Elevated Anterior Cingulate Cortex Glutamate and Lifetime History of Rapid Cycling

Marin Veldic M.D. (1)*, Vincent Millischer M.D. (2,3)*, John D. Port M.D., Ph.D. (4), Man Choi Ho Ph.D. (5), Yun-Fang Jia Ph.D. (5), Jennifer R Geske (6), Joanna M. Biernacka Ph.D. (1,6) Lena Backlund M.D. (2,3), Susan L. McElroy M.D. (7), David J. Bond M.D., Ph.D. (8), J Carlos Villaescusa Ph.D. (2,3), Michelle Skime (1), Doo-Sup Choi Ph.D. (1,5), Catharina Lavebratt Ph.D. (2,3), Martin Schalling M.D., Ph.D. (2,3), and Mark A. Frye M.D. (1)

*Equal contributions

Material and methods

Genotyping Cohort description

The BD cohort consisted of patients from the Mayo Clinic Individualized Medicine Biobank for Bipolar Disorder and patients recruited from the Unit of Affective disorders at Psychiatry Southwest, Karolinska University Hospital, Huddinge.

Patients in the Swedish cohort were consecutively recruited between 2003 and 2010, mostly from specialized outpatient clinics for affective disorders. Life-time manic and depressive symptoms were assessed. The assessment was based on interviews and medical records focusing on the most severe manic episode and performed by a psychiatrist specialized in bipolar disorder or by a trained psychiatric nurse. Manic and depressive symptoms were assessed using the modules for mania and depression in the Schedules for Clinical Assessment in Neuropsychiatry (SCAN) (Wing et al., 1990) or with the Affective Disorder Evaluation as has been described elsewhere (Ryden et al., 2009) Depressive symptoms were assessed according to DSM-IV. On the basis of these assessment patients were considered as fulfilling the diagnostic criteria for BD-I, BD-II, schizoaffective disorder, or not otherwise specified (NOS). The rapid cycling phenotype was also assessed. Venous blood DNA was obtained at the time of interview. In total from 726 unrelated BD patients were included. The genetic study was approved by the Regional Ethical Review Board in Stockholm in accordance with the Helsinki Declaration of 1975. All bipolar participants were in euthymic phase. In both studies all

individuals had full capacity to consent and the informed consent process was both verbal and written during a visit to a special trained psychiatric nurse.

Patients in the American cohort were recruited from the Mayo Clinic. Patients in the American genotyping cohort were recruited from the Individualized Medicine Biobank for Bipolar Disorder, a collaborative project between the Mayo Clinic, the Lindner Center of HOPE/University of Cincinnati, and the University of Minnesota, each with site-specific institutional review board approval. The written informed consent process was followed by a comprehension test questionnaire to ensure key points of study participation were understood. The biobank procedures for participant recruitment, clinical phenotyping, and biological specimen sampling, processing, and storage were published previously (Frye et al., 2015).

Eligible participants were adults (aged 18 years or older at enrollment) with clinical diagnoses of BD-I, BD-II, or schizoaffective disorder who were able to provide valid informed consent. Individuals who were actively psychotic or actively suicidal and needing psychiatric hospitalization were not approached initially for participation; however, patients who were initially excluded for these reasons were approached after acute psychiatric problems resolved.

Bipolar Biobank procedures included a detailed evaluation at baseline. BD-I and BD-II diagnoses, age of BD symptom onset, and comorbid psychiatric diagnoses were established using the Structured Clinical Interview for the DSM-IV (SCID). The baseline assessment included structured patient-rated and clinician-administered questionnaires to ascertain demographic variables, clinical variables, and illness characteristics (including lifetime history of rapid cycling).

Lifetime history of rapid cycling (RC) was defined as a self-reported history of having four or more distinct bipolar mood episodes in a 12 months period, with each episode separated by a return to baseline mood state for at least 2 months, or a switch to the opposite mood pole. Manic and hypomanic episodes were counted as being on the same mood pole.

Patient with MDD were derived from the PART study, a longitudinal population-based study in Stockholm County, Sweden (Hällström et al., 2003). PART includes data from 8613 randomly-selected Swedish nationals who have responded to an extensive questionnaire on mental health, work and relations twice (in two waves with a 3-yr interval), including the Major Depression Inventory (MDI). Individuals characterized as having depression were those diagnosed with major depression, mixed

anxiety depression or dysthymia, in at least one of the two PART waves. Depression was defined according to DSM-IV and was identified using the MDI.

The collection of the Swedish samples was approved by the Regional Ethical Review Board in Stockholm and informed consent was obtained from all participants.

MRI - Voxel positioning

For the midline anterior cingulate cortex (MACC) voxel, a reference slice was taken from an axial cut approximately 1 cm above the genu of the corpus callosum, demonstrating a continuous view of the anterior and posterior horns of the lateral ventricles. On this reference image, an 8-cm^3 voxel (2x2x2 cm) of predominantly gray (prefrontal) matter was centered on the frontal interhemispheric fissure. The posterior margin of the voxel was placed immediately anterior to the genu of the corpus callosum in an area corresponding to the pregenual ACC (Brodmann area 24a, 24b, and 32), as described by Vogt and Vogt (2003). For the left dorsolateral prefrontal cortex voxel (LDLPFC), a reference coronal oblique localizer slice was positioned on the sagittal anatomical images such that it was positioned perpendicular to the average plane of the corpus callosum, and the posterior margin of the slice was located immediately anterior to the anterior-most portion of the genu of the corpus callosum. On this reference image, an 8-cm3 voxel (2x2x2 cm) encompassing the LDLPFC was placed such that: 1) the superolateral corner of the voxel abutted, but did include, the skull, 2) the medial margin of the voxel excluded the medial frontal cortex, and 3) the voxel was placed as superiorly as possible given constraints 1 and 2. This positioning typically includes the superior frontal sulcus and large portions of the superior and middle frontal gyri, containing Brodmann's areas 9 and 46.

References

Frye, M.A., McElroy, S.L., Fuentes, M., Sutor, B., Schak, K.M., Galardy, C.W., et al. 2015. Development of a bipolar disorder biobank: differential phenotyping for subsequent biomarker analyses. Int J Bipolar Disord 3, 30.

Hällström T, Damström TK, Forsell Y, Lundberg I, Tinghög P (2003). The PART Study. A Population Based Study of Mental Health in the Stockholm County: Study Design. Phase l (1998–2000).

Ryden E, Thase ME, Straht D, Aberg-Wistedt A, Bejerot S, et al. (2009) A history of childhood attention-deficit hyperactivity disorder (ADHD) impacts clinical outcome in adult bipolar patients regardless of current ADHD. Acta Psychiatr

Vogt BA, Vogt L (2003). Cytology of human dorsal midcingulate and supplementary motor cortices. J Chem Neuroanat 26(4): 301-309.

Wing JK, Babor T, Brugha T, Burke J, Cooper JE, et al. (1990) SCAN. Schedules for Clinical Assessment in Neuropsychiatry. Arch Gen Psychiatry 47: 589–593.

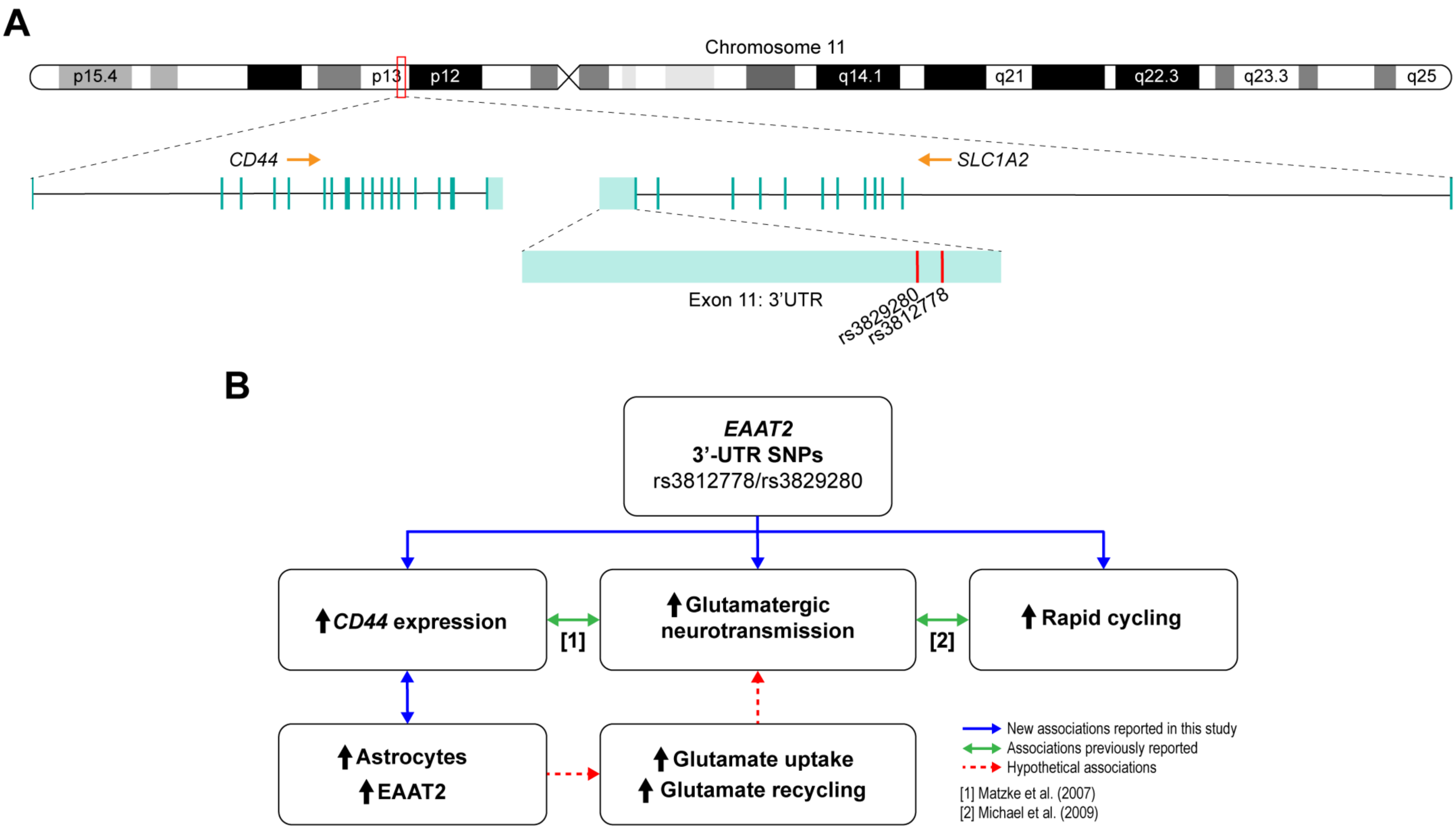

Supplementary Figure 1. Chromosomal location of *SLC1A2* and *CD44* and proposed Mechanism for the Impact of Genetic Variations in *SLC1A2* on Glutamatergic Neurotransmission and Rapid Cycling

(A) Single nucleotide polymorphisms (SNPs), rs3829280 and rs3812778, are located in the 3'-untranslated region of the excitatory amino acid transporter 2 (EAAT2) gene (*SLC1A2*) in chromosome 11. *CD44* is situated downstream of *SLC1A2*.

(B) Proposed mechanism for the impact of SNPs rs3829280 and rs3812778 on glutamatergic neurotransmission and rapid cycling.

Supplementary Table 1. Association of single nucleotide polymorphisms (SNP) with glutamate concentration measured by MR-spectroscopy in the left dorsolateral prefrontal cortex voxel (LDLPFC) and midline anterior cingulate cortex (MACC) of depressed patients. MAF = minor allele frequency. 2DJ = 2-dimensional J-resolved averaged PRESS sequence. TE80 = TE-optimized PRESS sequence. GLUL encoding glutamine synthetase, SLCA17 encoding excitatory amino acid transporter (EAAT) 5, SLC1A2 encoding EAAT2, SLC1A3 encoding EAAT1.

						LDLPFC 2DJ		LDLPFC TE80		MACC 2DJ		MACC TE80	
SNP	Minor	Major	MAF	Gene	Elements	Estimate	*p*	Estimate	*p*	Estimate	*p*	Estimate	*p*
rs1058111	A	G	0.48	*GLUL*	exon	-3.18	0.306	-0.59	0.082	-9.64	0.147	0.56	0.349
rs912901	C	G	0.17	*GLUL*	5'UTR	-3.65	0.348	-0.08	0.853	6.18	0.455	-0.80	0.239
rs62126236	G	A	0.27	*SLC17A7*	upstream 2kb	-3.72	0.306	0.00	0.998	-16.47	0.064	0.04	0.957
rs1042113	G	A	0.34	*SLC1A2*	exon	0.02	0.996	0.14	0.685	11.12	0.126	-1.05	0.067
rs1043101	G	A	0.31	*SLC1A2*	3'UTR	-1.39	0.683	-0.36	0.329	1.04	0.890	0.71	0.242
rs10768121	C	A	0.31	*SLC1A2*	3'UTR	-1.39	0.683	-0.36	0.329	1.04	0.890	0.71	0.242
rs11033046	T	A	0.31	*SLC1A2*	3'UTR	-1.39	0.683	-0.36	0.329	1.04	0.890	0.71	0.242
rs12361171	T	A	0.31	*SLC1A2*	3'UTR	-1.39	0.683	-0.36	0.329	1.04	0.890	0.71	0.242
rs3088168	G	A	0.31	*SLC1A2*	3'UTR	-1.39	0.683	-0.36	0.329	1.04	0.890	0.71	0.242
rs12294045	A	G	0.25	*SLC1A2*	3'UTR	4.14	0.253	-0.14	0.735	8.10	0.307	0.86	0.193
rs3812778	A	G	0.16	*SLC1A2*	3'UTR	1.00	0.799	0.24	0.580	22.63	0.004	0.97	0.159
rs3829280	T	A	0.16	*SLC1A2*	3'UTR	1.00	0.799	0.24	0.581	22.63	0.004	0.97	0.159
rs7936950	G	A	0.25	*SLC1A2*	3'UTR	3.14	0.343	0.36	0.326	-10.08	0.154	0.74	0.217
rs10742338	A	G	0.25	*SLC1A2*	3'UTR	3.14	0.343	0.36	0.326	-10.08	0.154	0.74	0.217
rs2229894	A	G	0.39	*SLC1A3*	3'UTR	-3.32	0.317	-0.38	0.324	-7.91	0.278	0.16	0.789

Supplementary Table 2. Effect sizes and p-values for all Affimetrix transcripts associated with *CD44* in different brain regions. Common homozygotes vs. Minor allele carriers. Data obtained from braineac.org. FDR = False discovery rate.

Affimetrix Transcript	All areas	Cerebellar cortex	Frontal cortex	Hippocampus	Medulla	Occipital cortex	Putamen	Substantia nigra	Temporal cortex	Thalamus	Intralobular white matter
3326636	0.06 (p=0.075)	-0.01 (p=0.9)	0 (p=0.987)	0.13 (p=0.301)	-0.03 (p=0.701)	-0.06 (p=0.552)	0.15 (p=0.165)	-0.01 (p=0.926)	0.23 (p=0.08)	0.21 (p=0.086)	0.05 (p=0.612)
3326653	**0.21 (p=0.027)**	0.29 (p=0.059)	0.23 (p=0.27)	0.17 (p=0.338)	0.26 (p=0.092)	0.27 (p=0.173)	0.37 (p=0.106)	**0.44 (p=0.021)**	0.21 (p=0.329)	0.19 (p=0.263)	0.19 (p=0.116)
3326658	0.18 (p=0.063)	0.22 (p=0.179)	0.35 (p=0.092)	0.08 (p=0.655)	0.23 (p=0.101)	0.3 (p=0.146)	0.24 (p=0.326)	**0.38 (p=0.046)**	0.1 (p=0.67)	0.16 (p=0.339)	0.08 (p=0.476)
3326666	**0.2 (p=0.047)**	**0.33 (p=0.049)**	0.36 (p=0.094)	0.04 (p=0.862)	0.25 (p=0.087)	0.34 (p=0.142)	0.28 (p=0.263)	0.38 (p=0.068)	0.04 (p=0.873)	0.2 (p=0.26)	0.19 (p=0.151)
3326669	**0.18 (p=0.03)**	0.15 (p=0.258)	0.25 (p=0.084)	0.18 (p=0.234)	0.21 (p=0.135)	0.15 (p=0.32)	0.34 (p=0.061)	**0.38 (p=0.02)**	0.04 (p=0.81)	0.19 (p=0.237)	0.17 (p=0.078)
3326682	0.05 (p=0.084)	0.04 (p=0.516)	0.07 (p=0.216)	0.11 (p=0.217)	0.03 (p=0.803)	0 (p=0.953)	0.06 (p=0.427)	0.18 (p=0.095)	0.01 (p=0.927)	0.04 (p=0.63)	0.02 (p=0.797)
3326683	**0.2 (p=0.013)**	**0.29 (p=0.005)**	0.22 (p=0.075)	**0.32 (p=0.045)**	0.25 (p=0.221)	0.01 (p=0.949)	**0.36 (p=0.009)**	0.25 (p=0.166)	0.1 (p=0.502)	0.26 (p=0.141)	0.23 (p=0.19)
3326684	**0.25 (p=0.004)**	0.1 (p=0.441)	**0.35 (p=0.012)**	0.23 (p=0.157)	0.36 (p=0.06)	0.13 (p=0.37)	**0.31 (p=0.028)**	**0.65 (p=0.001)**	**0.33 (p=0.04)**	0.04 (p=0.802)	0.33 (p=0.071)
3326685	**0.08 (p=0.05)**	0.1 (p=0.233)	0.16 (p=0.053)	0.13 (p=0.071)	0.1 (p=0.341)	0.01 (p=0.902)	-0.03 (p=0.659)	**0.21 (p=0.022)**	0.1 (p=0.241)	0.12 (p=0.182)	0.08 (p=0.409)
3326687	0.09 (p=0.141)	0.03 (p=0.709)	-0.05 (p=0.606)	0.04 (p=0.777)	0.13 (p=0.45)	0.04 (p=0.646)	**0.28 (p=0.001)**	0.27 (p=0.139)	0.07 (p=0.586)	0.02 (p=0.892)	**0.42 (p=0.003)**
3326694	0.07 (p=0.221)	0.18 (p=0.065)	0 (p=0.967)	0.12 (p=0.279)	0.25 (p=0.063)	0.16 (p=0.086)	0.1 (p=0.311)	0.04 (p=0.754)	0.01 (p=0.961)	-0.08 (p=0.509)	0.12 (p=0.282)
3326696	0.1 (p=0.099)	0.01 (p=0.93)	0.08 (p=0.42)	-0.03 (p=0.836)	0.14 (p=0.43)	0.18 (p=0.063)	0.17 (p=0.065)	0.15 (p=0.26)	0.13 (p=0.253)	0.19 (p=0.218)	0.22 (p=0.153)
3326705	0.06 (p=0.218)	0.05 (p=0.505)	0.03 (p=0.724)	0.08 (p=0.367)	0.04 (p=0.745)	0.05 (p=0.61)	0.02 (p=0.862)	0.13 (p=0.224)	0.12 (p=0.248)	0.17 (p=0.129)	0.06 (p=0.538)
3326712	**0.14 (p=0.023)**	0.2 (p=0.106)	**0.26 (p=0.033)**	0.17 (p=0.18)	0.26 (p=0.137)	0.04 (p=0.738)	0.11 (p=0.382)	0.03 (p=0.854)	0.07 (p=0.631)	0.09 (p=0.558)	**0.28 (p=0.05)**
3326714	0.09 (p=0.053)	0.1 (p=0.235)	0.04 (p=0.698)	0.11 (p=0.369)	0.1 (p=0.488)	0.15 (p=0.122)	0.11 (p=0.29)	0.13 (p=0.25)	0.03 (p=0.793)	0.14 (p=0.259)	0.09 (p=0.425)
3326717	0.17 (p=0.057)	0.08 (p=0.564)	0.1 (p=0.558)	0.12 (p=0.505)	0.27 (p=0.095)	0.26 (p=0.146)	0.39 (p=0.066)	**0.43 (p=0.018)**	0.17 (p=0.392)	0.17 (p=0.343)	0.15 (p=0.255)
3326720	0.05 (p=0.393)	**0.22 (p=0.037)**	0.14 (p=0.193)	0.13 (p=0.35)	-0.16 (p=0.308)	0.04 (p=0.748)	0.08 (p=0.465)	0.18 (p=0.209)	-0.01 (p=0.959)	0.1 (p=0.446)	-0.06 (p=0.642)
3326721	0.16 (p=0.08)	0.18 (p=0.159)	0.24 (p=0.115)	0.08 (p=0.625)	0.22 (p=0.195)	0.16 (p=0.28)	**0.39 (p=0.048)**	0.27 (p=0.144)	0.2 (p=0.219)	0.15 (p=0.364)	0.17 (p=0.211)
3326722	0.21 (p=0.073)	0.18 (p=0.38)	0.26 (p=0.276)	0.19 (p=0.382)	0.26 (p=0.147)	0.32 (p=0.173)	0.49 (p=0.089)	**0.5 (p=0.041)**	0.16 (p=0.531)	0.08 (p=0.7)	0.19 (p=0.197)
3326723	0.15 (p=0.068)	0.27 (p=0.084)	-0.08 (p=0.651)	-0.08 (p=0.668)	0.13 (p=0.487)	0.32 (p=0.075)	**0.36 (p=0.044)**	0.1 (p=0.647)	0.26 (p=0.184)	0.12 (p=0.565)	0.32 (p=0.068)
3326726	0.16 (p=0.114)	0.27 (p=0.091)	0.13 (p=0.496)	0.11 (p=0.588)	0.28 (p=0.104)	0.2 (p=0.305)	0.32 (p=0.176)	0.4 (p=0.052)	0.19 (p=0.369)	0.15 (p=0.41)	0.12 (p=0.392)
3326728	**0.2 (p=0.031)**	0.2 (p=0.161)	0.22 (p=0.199)	0.1 (p=0.575)	0.26 (p=0.087)	0.25 (p=0.14)	**0.43 (p=0.038)**	**0.52 (p=0.005)**	0.16 (p=0.395)	0.13 (p=0.427)	0.11 (p=0.424)
3326729	0.21 (p=0.039)	0.17 (p=0.262)	0.2 (p=0.282)	0.22 (p=0.304)	0.28 (p=0.086)	0.17 (p=0.375)	**0.53 (p=0.021)**	0.47 (p=0.03)	0.19 (p=0.363)	0.18 (p=0.332)	0.11 (p=0.492)
t3326635	**0.15 (p=0.02)**	**0.16 (p=0.046)**	0.15 (p=0.139)	0.13 (p=0.266)	0.19 (p=0.106)	0.16 (p=0.133)	**0.27 (p=0.028)**	**0.28 (p=0.023)**	0.14 (p=0.27)	0.13 (p=0.276)	0.18 (p=0.069)

Legend

- FDR ≤ 0.1
- 0.10 < FDR ≤ 0.15
- 0.15 < FDR ≤ 0.20
- **Bold: p < 0.05**

Supplementary Table 3. GWAVA results. Output from the GWAVA annotation tool for all SNPs in perfect LD with rs3812778/rs3829280. Region score, TSS score and Unmatched score are scores from 3 different versions of the classifier, which are all in the range 0-1. Higher scores indicate that variants are predicted to be more likely to be functional. The other columns show the scores for the underlying annotations used to compute these scores.

ID	Chromosome	Position	Region score	TSS score	Unmatched score	Average GERP	Average DAF	Average het	GERP	%GC	TSS distance	SS distance
rs67384276	11	35,270,584	0.27000	0.14000	0.08000	0.10341	0.00228	0.00188	0.11600	46.5%	30107	-5771
rs56193087	11	35,271,028	0.20000	0.30000	0.13000	-1.07033	0.00291	0.00249	-3.04000	53.5%	30551	-5327
rs12360706	11	35,276,059	0.57000	0.80000	0.79000	0.58727	0.00018	0.00033	1.23000	53.5%	-27300	-296
rs3829280	11	35,276,723	0.50000	0.64000	0.70000	-0.25067	0.00133	0.00076	-0.37600	43.6%	-26636	365
rs3812778	11	35,277,270	0.42000	0.68000	0.68000	-0.09887	0.00135	0.00082	0.66700	43.6%	-26089	912
rs4508184	11	35,282,096	0.58000	0.61000	0.66000	0.53791	0.00033	0.00057	3.25000	49.5%	-21263	-416
rs1570216	11	35,282,334	0.62000	0.67000	0.69000	2.74575	0.00035	0.00062	-7.09000	33.7%	-21025	-178
rs10836358	11	35,287,500	0.37000	0.18000	0.11000	0.04928	0.00022	0.00040	1.39000	40.6%	-15859	192

ID	CEBPB	DNase	EXON	FAIRE	H3K27ac	H3K27me3	H3K36me3	H3K4me1	H3K4me2	H3K9ac	INTRON	REP	STAT3	TRAN	UTR3	WEAK_ENH	dnase_fps	repeat
rs67384276	1	1	-	1	-	1	-	-	-	-	-	3	-	1	-	-	-	1
rs56193087	-	-	-	-	-	1	-	-	-	-	-	6	-	-	-	-	-	1
rs12360706	-	-	1	1	-	2	-	1	-	-	-	5	-	1	1	-	-	-
rs3829280	-	-	1	-	-	2	-	1	-	-	1	5	-	1	1	-	-	-
rs3812778	-	2	1	-	-	2	-	-	-	-	1	5	-	1	1	-	-	-
rs4508184	-	1	1	2	4	1	-	2	4	1	1	4	-	1	1	1	2	-
rs1570216	-	63	1	1	4	1	-	2	4	1	1	5	3	-	1	1	21	-
rs10836358	-	-	-	1	-	2	1	1	-	-	1	4	-	1	-	-	-	1

Supplementary Table 4. Effects of Genetic Variations in SLC1A2 (rs3812778) and P2RX7 (rs2230912) on Rapid Cycling Using Logistic Regression.

Model 1. Additive model of the effects of the minor alleles of rs3812778 and rs2230912 on rapid cycling. Patients with rapid cycling BD versus patients with non-rapid cycling BD, logistic regression.
Model 2. Interactive model of the effects of the minor alleles of rs3812778 and rs2230912 on rapid cycling. Patients with rapid cycling BD versus patients with non-rapid cycling BD, logistic regression.
Model 3. Additive model of the effects of the minor alleles of rs3812778 and rs2230912 on rapid cycling. Patients with rapid cycling BD versus combined patients with non-rapid cycling BD and unipolar depression, logistic regression.
Model 4. Interactive model of the effects of the minor alleles of rs3812778 and rs2230912 on rapid cycling. Patients with rapid cycling BD versus combined patients with non-rapid cycling BD and unipolar depression, logistic regression.
OR = Odds Ratio; CI = Confidence Interval

	**Model 1**		**Model 2**		**Model 3**		**Model 4**	
	OR (95% CI)	*p*	OR (95% CI)	*p*	OR (95% CI)	*p*	OR (95% CI)	*p*
**rs3812778**								
Minor allele carriers	1.36 (1.05-1.76)	0.018	1.54 (1.14-2.10)	0.005	1.37 (1.08-1.72)	0.008	1.56 (1.18-2.05)	0.002
Common allele homozygotes	Ref		Ref		Ref		Ref	
**rs2230912**								
Minor allele carriers	0.92 (0.72-1.17)	0.50	1.03 (0.77-1.36)	0.86	0.88 (0.70-1.09)	0.25	0.98 (0.76-1.27)	0.901
Common allele homozygotes	Ref		Ref		Ref		Ref	
**Interaction**								
rs3812778 x rs2230912			0.65 (0.37-1.14)	0.13			0.64 (0.39-1.07)	0.091
